# Evaluation of the Repeatability and Reproducibility of the Results of Lymph Node Cytology for *Leishmania* Amastigotes in Dogs Without Leishmaniosis and in Dogs with Leishmaniosis Before, During, and After Treatment

**DOI:** 10.3390/vetsci12111073

**Published:** 2025-11-08

**Authors:** Adamantia Pseftogka, Elisabeta Samuel (Badulescu), Manolis K. Chatzis, Eleni Kouklaki, Mathios E. Mylonakis, Manolis N. Saridomichelakis

**Affiliations:** 1 Clinic of Medicine, Faculty of Veterinary Science, University of Thessaly, Trikalon Str. 224, 43132 Karditsa, Greece; adpseftogka@outlook.com.gr (A.P.); elisa.badulescu@artvetderm.ro (E.S.); mchatzis@vet.uth.gr (M.K.C.); 2 Artvetderm, Strada General Dimitrie Salmen 2, 021272 Bucharest, Romania; 3 Companion Animal Clinic, School of Veterinary Medicine, Faculty of Health Sciences, Aristotle University of Thessaloniki, Stavrou Voutyra 11, 54627 Thessaloniki, Greece; elkouklaki1@gmail.com (E.K.); mmylonak@vet.auth.gr (M.E.M.)

**Keywords:** canine, cytology, dog, diagnosis, *Leishmania infantum*, leishmaniosis, lymph node, repeatability, reproducibility

## Abstract

**Simple Summary:**

A diagnostic test should be sensitive (positive in most subjects with the condition under investigation), specific (negative in most subjects without the condition), repeatable (results should be the same when the same person performs the test more than once), and reproducible (results should be the same when the same person performs the test in different samples from the same subject and when different persons perform the test using the same sample). We have already shown that cytologic examination of lymph nodes is sensitive (93%) and specific (100%) for the detection of the organism in dogs with leishmaniosis, a severe and widespread parasitic disease. In this study we obtained two lymph node samples from each dog with or without the disease, and three blinded examiners evaluated them twice. We found that the qualitative (positive or negative) and semiquantitative (the number of parasites) repeatability (agreement when the same examiner evaluated twice the same sample), inter-sample reproducibility (agreement when the same examiner evaluated the two lymph nodes from the same dog), and interobserver reproducibility (agreement between the results of the same sample when evaluated by different examiners) are substantially higher than expected by chance.

**Abstract:**

Lymph node (LN) cytology is sensitive for the detection of parasites in dogs with leishmaniosis (CanL) and is used for the semiquantitative measurement of parasitic burden. The aim of this study was to investigate the repeatability and reproducibility of the qualitative (detection of *Leishmania* amastigotes) and semiquantitative (measurement of parasitic burden using a logarithmic scale) results of LN cytology. Three experienced examiners evaluated, blindly and independently of each other, two LN smears from each of the 53 dogs, including dogs without CanL (*n* = 40 smears), dogs with CanL (*n* = 32 smears), and dogs during or after treatment of CanL (*n* = 40 smears). Cohen’s kappa coefficient (κ) indicated substantial-to-good agreement of the qualitative results when the same examiner evaluated the same smear twice (intraobserver repeatability; κ = 0.75–0.964), when the same examiner evaluated the two smears from the same dog (inter-sample reproducibility; κ = 0.626–0.964), and when different examiners evaluated the same smears (interobserver reproducibility; κ = 0.626–0.893). For the semiquantitative results, allowing a one-point difference in each pair of measurements, agreement was also substantial to good with κ values of 0.952–1 (intraobserver repeatability), 0.732–0.935 (inter-sample reproducibility), and 0.804–0.941 (interobserver reproducibility). High repeatability and reproducibility, in addition to high sensitivity, indicate that LN cytology is useful for diagnosis and treatment monitoring of CanL.

## 1. Introduction

According to current guidelines, the diagnosis of canine leishmaniosis (CanL) due to *Leishmania infantum* should be based on compatible clinical signs and/or clinicopathologic abnormalities, exclusion of major differentials, increased serum concentration of anti-*Leishmania* IgG antibodies, and demonstration of infection by detection of the parasite or parasitic DNA [1]. Moreover, lymph nodes (LNs) are among the tissues with high parasitic density where evaluation of parasitic burden by semiquantitative cytology and/or quantitative PCR (qPCR) is mandatory for treatment monitoring and for informing the decision for treatment discontinuation [1,2,3]. Sampling of peripheral LNs is less invasive compared to other tissues with a high parasitic burden like bone marrow or the spleen and can be easily performed in dogs with lymphadenomegaly, one of the most common clinical signs of CanL [1,4].

In addition to absolute specificity when performed by experienced examiners, LN cytology has been shown to have a diagnostic sensitivity of 93% in dogs with leishmaniosis, provided that 1000 oil immersion fields (OIFs) of high-quality smears are examined [5]. Additionally, when amastigote numbers are expressed on a logarithmic scale [6], a decline in parasitic burden during effective treatment of CanL has been demonstrated [7].

High repeatability and reproducibility are necessary for a diagnostic test to be considered clinically useful [8,9]. To the best of our knowledge, these attributes of LN cytology for both the presence (qualitative results) and the semiquantitative counting of *Leishmania* amastigotes have not been reported.

The aim of this study was to investigate the repeatability, inter-sample reproducibility, and interobserver reproducibility of the qualitative and semiquantitative results of LN cytology for *Leishmania* amastigotes.

## 2. Materials and Methods

The study protocol was approved by the Animal Ethics Committee of the Faculty of Veterinary Science, University of Thessaly, Greece (license No 166/20.10.2023). The handling of the dogs complied with the European Communities Council directive 2010/63/EU and state legislation. The owners signed an informed consent form for the participation of their dogs in the study if LN sampling was not part of the standard-of-care diagnostic work-up.

Paired peripheral LN fine-needle non-aspiration smears were prepared from 20 dogs without CanL (group A), 16 dogs with CanL before treatment (group B), and 17 dogs during or after treatment of CanL (group C). Since the same dogs from group C could be included more than once in subsequent re-examinations, the total number of samplings from group C dogs was 20.

Inclusion criteria for group A dogs were having at least two peripheral LNs that were palpable and easy to puncture, exclusion of CanL based on negative serology (the Snap^®^ Leishmania Test; Idexx and/or the indirect immunofluorescence antibody test), and routine LN cytology; the latter was performed in representative OIFs to investigate the cause of lymphadenomegaly when present (e.g., lymphoma, metastatic neoplasia, lymphadenitis, and reactive processes). Group B dogs were diagnosed with CanL based on clinical signs, clinicopathologic abnormalities, exclusion of major differentials, and positive results of serology and of routine LN cytology. Group C dogs had been diagnosed with CanL and were under treatment with drugs with direct anti-*Leishmania* activity and/or with immunomodulators [1], or treatment had been discontinued for a period of up to 6 months.

The two easiest-to-puncture peripheral LNs of each dog were selected for sampling, and their size was subjectively scored as normal or mildly, moderately, or severely enlarged [7]. A fine-needle non-aspiration technique, using 1-inch 21 G needles, was used. The specimen was expelled onto a glass slide, and the squash technique was used to prepare a smear that was subsequently air-dried and stained with Diff-Quik (Merck; Darmstadt, Germany). Therefore, a total of 20 × 2 = 40 LN smears were prepared from group A and group C dogs, and a total of 16 × 2 = 32 smears were prepared from group B dogs.

**Figure 1:**
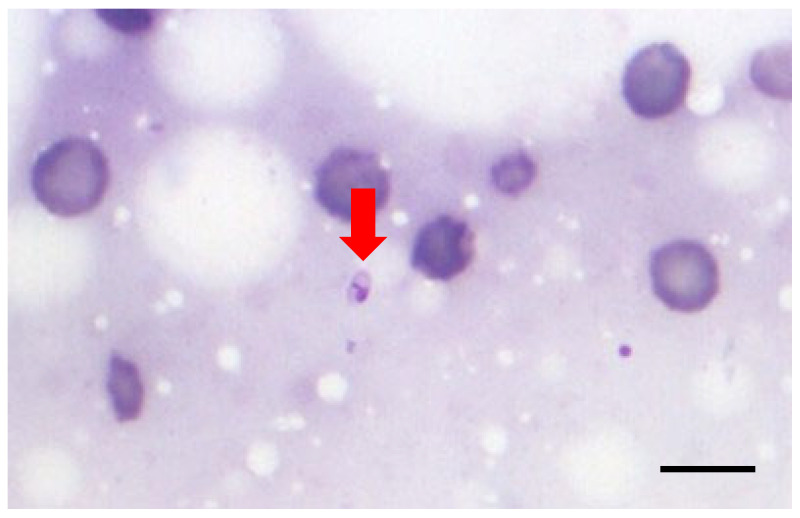
One *Leishmania* amastigote (arrow) among red blood cells. The key morphological features (plasma membrane, nucleus, and kinetoplast that has a more condensed staining than the nucleus) are clearly visible. The three-dimensional orientation of this amastigote is optimal because the long axis, the nucleus, and the kinetoplast are parallel to the surface of the glass slide. Magnification: 1000×; bar: 10 μm.

After all 112 LN smears were collected, their identity was masked, and each of them was coded with a random number (https://www.calculator.net/random-number-generator.html; accessed on 2 December 2024) by an investigator (MNS) who was not involved in their subsequent cytologic examination. Three investigators examined the smears twice (1st and 2nd examination round with a minimum period of 2 weeks between them) independently of each other at 1000× magnification for the presence and semiquantitative counting of *Leishmania* amastigotes. The examiners included a 3rd year resident of the European College of Veterinary Dermatology (ECVD; AP-examiner #1), a diplomate of ECVD (ESB-examiner #2), and a postdoctoral clinician with a PhD in leishmaniosis (MKC-examiner #3). Only OIFs with at least one nucleated cell were examined; well-stained OIFs with moderate cellularity and minimal blood contamination and cells in a monolayer were preferred, and identification of *Leishmania* amastigotes was based on a clearly visible plasma membrane, nucleus, and kinetoplast, with the staining of the latter being more intense compared to the nucleus due to the more compact DNA [1,5] (Figure 1). The parasitic burden was expressed using the following logarithmic scale: +6: >1000 amastigotes/10 OIFs; +5: 100–999 amastigotes/10 OIFs; +4: 10–99 amastigotes/10 OIFs; +3: 1–9 amastigotes/10 OIFs; +2: 1–9 amastigotes/100 OIFs; +1: 1–9 amastigotes/1000 OIFs; and 0: no amastigotes/1000 OIFs [6].

Based on the results of previous studies, it was assumed that approximately 60% of group A dogs would be subclinically infected by *L. infantum* and that LN cytology would be positive in 25% of them [5], resulting in approximately 6/40 positive group A smears. Since *Leishmania* amastigotes had been found during routine LN cytology, it was expected that all 32 group B smears would be positive, and a 50% (20/40) positivity rate among group C smears was assumed. Therefore, it was expected that approximately 58/112 (51.8%) of the smears would be positive for *Leishmania* amastigotes, resulting in a lack of bias for the blinded examiners (i.e., before the examination of each smear, they knew that there were almost equal chances to be positive or negative).

The distribution of continuous data was tested with the Shapiro–Wilk test. Normally distributed data are presented as the mean ± standard deviation, whereas data not following a normal distribution and ordinal data are presented as the median (range).

For the qualitative (finding or not amastigotes) and semiquantitative (only positive pairs of slides were included in their analysis to avoid artificially increasing concordance due to the numerous negative group A, and to a lower degree group C, slides) results, Cohen’s kappa coefficient (κ) was used to estimate the agreement between (a) the two examinations (1st and 2nd examination rounds) of the same slide by the same examiner (intraobserver repeatability), (b) the two slides from different LNs of the same dog for the same examiner and the same examination round (inter-sample reproducibility), and (c) each pair of examiners for the same slide and the same examination round (interobserver reproducibility). Analyses of the semiquantitative results were performed using weighted (quadratic weights) Cohen’s kappa, and they were repeated after both values of each pair of results differing by one point were transformed to their average (e.g., if a pair of results had values of +3 and +4, both were transformed to +3.5). The reason for this tolerance for a one-point difference is the inherent drawback of the logarithmic scale of Chulay and Bryceson [6], where each pair of consecutive scores can differ by a single amastigote.

Agreement was considered poor if κ ≤ 0.2, fair if 0.21 ≤ κ ≤ 0.4, moderate if 0.41 ≤ κ ≤ 0.6, substantial if 0.61 ≤ κ ≤ 0.8, and good if κ > 0.8 [9]. All analyses were performed using IBM SPSS 29.0.1 for Windows, and the level of significance was 5%.

## 3. Results

### 3.1. Dogs and Lymph Nodes

The 53 dogs (20 group A, 16 group B, and 17 group C) included 22 (41.5%) intact males, 7 (13.2%) castrated males, 8 (15.1%) intact females, and 13 (24.5%) spayed females; sex and neuter status were not recorded for 3/53 dogs. They belonged to 15 different breeds [English setter, Greek hound, and Pitbull terrier (*n* = 3, each); Belgian shepherd and Yorkshire terrier (*n* = 2, each); and American bullterrier, Doberman pincher, Dogo Argentino, English bulldog, German shorthaired pointer, German wirehaired pointer, Jack Russel terrier, Labrador retriever, Schnauzer, and Spitz (*n* = 1, each)] or were cross-breeds (*n* = 29); the breed was not recorded for 1/53 dogs. Their average age was 7.3 ± 3.5 years, and their average body weight was 21.4 ± 11 kg. The distribution of sex and neuter status in each group is presented in Table A1, and the age and body weight are presented in Table A2.

The 112 sampled LNs (40 group A, 32 group B, and 40 group C) included 73 (65.2%) popliteal, 30 (26.8%) prescapular, 8 (7.1%) submandibular, and 1 (0.9%) inguinal (Table A3). Their sizes were subjectively graded as normal (16–14.3%), mildly enlarged (51–45.5%), moderately enlarged (31–27.7%), or severely enlarged (14–12.5%) (Table A4).

### 3.2. Qualitative Results (Finding or Absence of Leishmania Amastigotes)

The prevalence of finding *Leishmania* amastigotes for each examiner and examination round is presented in Table 1.

The intraobserver repeatability of the qualitative results was substantial [κ = 0.74, 95% confidence interval (CI): 0.613–0.867; examiner #1] or good [κ = 0.964 (95% CI: 0.915–1) for examiner #2 and κ = 0.802 (95% CI: 0.692–0.912) for examiner #3].

The inter-sample reproducibility of the qualitative results for the two slides from different LNs of the same dog was substantial [κ = 0.626 (95% CI: 0.418–0.834) and κ = 0.779 (95% CI: 0.612–0.946) for the first and second examination rounds, respectively, of examiner #1] or good [κ = 0.893 (95% CI: 0.776–1) and κ = 0.964 (95% CI: 0.895–1) for the first and second examination rounds, respectively, of examiner #2; κ = 0.853 (95% CI: 0.714–0.992) and κ = 0.821 (95% CI: 0.672–0.97) for the first and second examination round, respectively, of examiner #3].

The interobserver reproducibility of the qualitative results was substantial (4/6) or good (2/6) (Table 2).

### 3.3. Semiquantitative Results

The parasitic burden for the slides where *Leishmania* amastigotes were found, for each examiner and in each examination round, is presented in Table A5.

The intraobserver repeatability of the semiquantitative results was good [κ = 0.803 (95% CI: 0.683–0.923) for examiner #1, κ = 0.912 (95% CI: 0.845–0.979) for examiner #2, and κ = 0.887 (95% CI: 0.82–0.954) for examiner #3]. When each pair of results differed by one point, both values were transformed to their average, and repeatability remained good for all examiners [κ = 0.948 (95% CI: 0.889–1) for examiner #1, κ = 0.905 (95% CI: 0.727–1) for examiner #2, and κ = 1 for examiner #3)].

The inter-sample reproducibility of the semiquantitative results for the two slides from different LNs of the same dog was moderate [2/6; κ = 0.569 (95% CI: 0.324–0.814) and κ = 0.568 (95% CI: 0.339–0.797) for the first examination round of examiner #1 and the second examination round of examiner #2, respectively], substantial [3/6; κ = 0.635 (95% CI: 0.439–0.831) for the first examination round of examiner #2, κ = 0.681 (95% CI: 0.524–0.838) for the first examination round of examiner #3, and κ = 0.72 (95% CI: 0.579–0.861) for the second examination round of examiner #3], or good [κ = 0.808 (95% CI: 0.702–0.914) for the second examination round of examiner #1]. When each pair of results differed by one point, both values were transformed to their average, and the inter-sample reproducibility became substantial [3/6; κ = 0.734 (95% CI: 0.583–0.885) for the first examination round of examiner #1, κ = 0.721 (95% CI: 0.429–1) for the first examination round of examiner #2, and κ = 0.774 (95% CI: 0.535–1) for the second examination round of examiner #2] or good [3/6; κ = 0.833 (95% CI: 0.647–1) for the second examination round of examiner #1, κ = 0.956 (95% CI: 0.874–1) for the first examination round of examiner #3, and κ = 0.854 (95% CI: 0.715–0.993) for the second examination round of examiner #3].

The interobserver reproducibility of the semiquantitative results was substantial (Table 3). When each pair of results differed by one point, both values were transformed to their average, and the interobserver reproducibility was substantial (2/6) or good (4/6) (Table 4)

## 4. Discussion

We showed that, under the conditions of this study, the intraobserver repeatability, inter-sample reproducibility, and interobserver reproducibility of LN cytology for the detection of *Leishmania* amastigotes and the measurement of parasitic burden are typically good (i.e., κ > 0.8) or, in the worst case, substantial (i.e., 0.61 ≤ κ ≤ 0.8). These results, in combination with the high sensitivity and specificity of this test [5], imply that LN cytology is valuable for the diagnosis and treatment monitoring of CanL.

There are multiple factors affecting the performance of LN cytology for the diagnosis of infection by *L. infantum* and the measurement of parasitic burden, and these factors are related to the examiners (experience and bias), the smears (quality and the parasitic burden), the cytologic procedure (selection and the number of examined OIFs), and the clinical interpretation of the results.

All three examiners could be subjectively considered “experienced”, but their level of experience at the beginning of the study varied according to their specialization level, their academic status, and their previous participation in similar research projects. In particular, examiner #1 (a third-year ECVD resident) had examined fewer LN smears from dogs suspected of CanL compared to examiner #2 (an ECVD diplomate), whereas examiner #3 had been routinely involved in examining clinical samples for amastigotes, in addition to the blind cytologic examination for *Leishmania* amastigotes of LN, bone marrow, skin, and conjunctival smears from 41 infected and 59 non-infected cats [10]. However, if examiner skills follow a learning curve ending in a plateau, it is possible that the experience of examiner #1, and perhaps of examiner #2, gradually increased during the first examination round and maximized before or during the second examination round. This may explain, for example, why the inter-sample reproducibility of the semiquantitative results of examiner #1 was much higher in the second examination round (κ = 0.808 and κ = 0.833 without and with tolerance for a one-point difference, respectively) compared to the first examination round (κ = 0.569 and κ = 0.734 without and with tolerance for a one-point difference, respectively).

This study was designed in such a way that, before starting the examination of each smear, examiners had zero bias in favor or against the detection of *Leishmania* amastigotes. To ensure this, many (*n* = 112) masked smears were prospectively collected, and the examiners knew that their results would have no clinical consequences for the patients, and they were aware of the preliminary calculations showing that the chances for each smear to be positive or negative were approximately equal. At the end of the first examination round, this equality estimation was proven to be quite accurate since, depending on the examiner, 55/112 (49.1%) and up to 68/112 (60.7%) of the slides were positive (Table 1); therefore, the lack of bias was not compromised before the second examination round. Finally, the large number of samples and the minimum period of 2 weeks between the two examination rounds were safeguards against recall bias. Subsequently, our results on repeatability and reproducibility reflect the common clinical scenario when LN smears are sent to external diagnostic laboratories and examined by an unbiased, well-trained clinical pathologist, as well as the scenario of blind examination of smears in research studies.

The quality of the 112 LN smears was subjectively considered “adequate” but not necessarily “ideal”, and it differed between smears obtained from the same dog and among smears from different dogs. Differences in the degree of blood contamination, cellularity, spreading of nucleated cells, and staining intensity can affect the measured parasitic burden (semiquantitative results) and, especially if the latter is low (e.g., a +1 score that corresponds to fewer than 10 amastigotes per 1000 OIFs), may also affect the qualitative results. This, in conjunction with the different optical fields examined when each smear was evaluated by the same examiner and by different examiners, partially accounts for the non-perfect repeatability and reproducibility of the results. Repeatability and reproducibility could have probably been higher if less-than-“ideal” smears had been discarded and if a pre-marked area of each smear had been used for cytology. However, in this case, the results would not have been representative of real-life conditions in both the clinical and research settings. The use of an alternative technique, called liquid-based cytology, where the LN samples are incubated with a preservative fluid and stained with Papanikolaou stain, would improve the quality of the smears, but this would likely be achieved at the expense of test sensitivity [11].

As already explained, smears with a very low parasitic burden, which is typical of group A dogs (Table A5), were the major source of discordant test results, as observed in bone marrow and spleen smears from humans with visceral leishmaniosis [12]. This could have been avoided by examining 100 instead of 1000 OIFs because all +1 results would have become negative, while all negative results would have remained negative. Also, the examination of 100 OIFs is much more time-efficient. However, if examination stops when no amastigotes are found in the first 100 OIFs, the sensitivity of this test for the detection of amastigotes in dogs with the disease decreases significantly from 93% to 84% [5]. The same would have happened if parasitic burden had been expressed in Leishman Donovan Units, which are the number of amastigotes per 1000 nucleated cells [13,14]; considering that an average OIF with the cells in a monolayer contains 10–20 nucleated cells, the examination would have stopped after the first 50–100 OIFs. In addition, the procedure would have been less practical due to the need to count the number of nucleated cells in each OIF, regardless of the presence of absence of amastigotes.

The way that the results of cytology should be interpreted in the clinical setting differs between the qualitative and semiquantitative results. Qualitative results (presence or absence of at least one amastigote per 1000 OIFs) provide an important piece of information for the diagnosis of CanL in dogs with compatible clinical signs and/or laboratory abnormalities [1], but they are not useful for the diagnosis of subclinical infection [1] (low sensitivity [5]) or for treatment monitoring (effective treatment does not completely eliminate the parasite [1]). On the contrary, semiquantitative results are not useful for the diagnosis of CanL (overlapping parasitic burden in dogs with the disease and subclinically infected ones [5] [Table A5]) but are important for treatment monitoring [1]. A drawback of the logarithmic scale of Chulay and Bryceson [6] is that each pair of consecutive scores can differ by a single amastigote. As an example, if nine amastigotes are found in the first 10 OIFs, the parasitic burden will be scored +3, whereas if one more parasite had been found (i.e., 10 amastigotes/10 OIFs), the score would increase to +4. This means that each one-point difference probably has minimal clinical importance. For this reason, semiquantitative results were reanalyzed after both values of each pair of results differing by one point were transformed to their average, and repeatability and reproducibility were almost always good (κ > 0.8), and in one case, they were perfect (κ = 1).

To the best of our knowledge, there is no published information on the repeatability and reproducibility of cytology for *Leishmania* amastigotes in LNs or any other tissues or organs of dogs. However, findings somewhat similar to ours, interobserver reproducibility for the detection of *Leishmania donovani* amastigotes (qualitative results) in spleen and bone marrow smears from humans with visceral leishmaniasis, are reported as “perfect” when the parasitic burden is +2 or higher, whereas when it was +1, there was “considerable discrepancy” between the two examiners; unfortunately numerical results were not provided [12].

Molecular methods represent an alternative to cytology for the confirmation of infection and the measurement of parasitic burden [1]. It is well known that the diagnostic performance of molecular tests depends heavily on the methodology, including the selected DNA target, and that discrepant qualitative results are not uncommon when examining the same samples with different PCRs, or even with the same PCR [15,16,17,18,19,20,21]. Reported κ values for the intra-assay repeatability of the qualitative results of PCR (repeated testing of the same samples in the same laboratory using the same technique) range from 0.6 to 1 [22], whereas inter-assay reproducibility (testing the same samples in different laboratories using the same technique) varies from 0.9 to 1 [22]. Discordant qualitative results between the right and left conjunctival scrapes from the same dogs (inter-sample reproducibility) have also been reported [23,24,25,26]; although the κ values are not published, for those studies where descriptive results are provided in detail, we calculated that the inter-sample reproducibility between the right and left conjunctival scrapes were 0.314 [25] or 0.4 [26] for conventional PCR (fair agreement), 0.571 (moderate agreement) for nested PCR [25], and −0.154 (no agreement) for qPCR [23]. Finally, a variability range of 7.5–19% in the quantification of the parasites with the same qPCR in the same samples is reported [27]. Although a direct comparison between these results for molecular methods and our results is not possible, it seems that the intra-assay repeatability, inter-sample reproducibility, and inter-assay reproducibility of LN cytology for the presence of *Leishmania* amastigotes and for the measurement of parasitic burden, under the conditions of the current study, are at least comparable to PCR.

## 5. Conclusions

The intraobserver repeatability, inter-sample reproducibility, and interobserver reproducibility of LN cytology for the detection of *Leishmania* amastigotes and semiquantitative measurement of parasitic burden are typically good when performed by experienced examiners. An examination of one LN smear from dogs suspected of the disease (for detection of amastigotes) and from dogs under and after treatment of CanL (for measurement of parasitic burden) is adequate in terms of result reproducibility. Serial LN smears obtained over time from dogs under and after treatment can be examined by different experienced examiners because of the reproducibility of the results.

## Figures and Tables

**Table 1:** Prevalence of positive cytology for *Leishmania* amastigotes in peripheral lymph nodes (LNs) of dogs without CanL (group A), dogs with CanL before treatment (group B), and dogs during or after treatment of CanL (group C) when examined twice (1st and 2nd examination rounds) by three investigators (examiners #1, #2, and #3).

	Group A (*n* = 40 LNs)	Group B (*n* = 32 LNs)	Group C (*n* = 40 LNs)
Examiner #1			
1st round	7/40 (17.5%)	31/32 (96.9%)	30/40 (75%)
2nd round	3/40 (7.5%)	32/32 (100%)	31/40 (77.5%)
Examiner #2			
1st round	1/40 (2.5%)	31/32 (96.9%)	23/40 (57.5%)
2nd round	1/40 (2.5%)	32/32 (100%)	24/40 (60%)
Examiner #3			
1st round	3/40 (7.5%)	32/32 (100%)	31/40 (77.5%)
2nd round	1/40 (2.5%)	32/32 (100%)	26/40 (65%)

**Table 2:** Cohen’s kappa coefficient (κ) of the interobserver reproducibility of the qualitative (positive or negative) results of lymph node cytology for *Leishmania* amastigotes in dogs without CanL, dogs with CanL before treatment, and dogs during or after treatment of CanL for each examiner (examiners #1, #2, and #3) and the same examination round (1st and 2nd examination rounds).

Pairs of Examiners	1st Examination Round	2nd Examination Round
#1 and #2	0.626 (95% CI: 0.485–0.767)	0.767 (95% CI: 0.649–0.885)
#1 and #3	0.703 (95% CI: 0.568–0.838)	0.838 (95% CI: 0.738–0.938)
#2 and #3	0.769 (95% CI: 0.653–0.885)	0.893 (95% CI: 0.809–0.977)

CI: confidence interval.

**Table 3:** Cohen’s kappa coefficient (κ) of the interobserver reproducibility of the semiquantitative results of lymph node cytology for *Leishmania* amastigotes in dogs without CanL, dogs with CanL before treatment, and dogs during or after treatment of CanL for each examiner (examiners #1, #2, and #3) and the same examination round (1st and 2nd examination rounds).

Pairs of Examiners	1st Examination Round	2nd Examination Round
#1 and #2	0.739 (95% CI: 0.61–0.868)	0.745 (95% CI: 0.637–0.853)
#1 and #3	0.763 (95% CI: 0.636–0.89)	0.79 (95% CI: 0.67–0.88)
#2 and #3	0.702 (95% CI: 0.561–0.843)	0.737 (95% CI: 0.621–0.853)

CI: confidence interval.

**Table 4:** Cohen’s kappa coefficient (κ) of the interobserver reproducibility of the semiquantitative results of lymph node cytology for *Leishmania* amastigotes in dogs without CanL, dogs with CanL before treatment, and dogs during or after treatment of CanL, for each pair of examiners (examiners #1, #2, and #3) and the same examination round (1st and 2nd examination round) after each pair of results differing by one point was transformed to their average.

Pairs of Examiners	1st Examination Round	2nd Examination Round
#1 and #2	0.745 (95% CI: 0.606–0.884)	0.765 (95% CI: 0.618–0.912)
#1 and #3	0.888 (95% CI: 0.808–0.968)	0.893 (95% CI: 0.785–1)
#2 and #3	0.853 (95% CI: 0.733–0.973)	0.91 (95% CI: 0.818–1)

CI: confidence interval.

## Data Availability

The original contributions presented in this study are included in the article. Further inquiries can be directed to the corresponding author.

## Abbreviations

The following abbreviations are used in this manuscript:

CanL	Canine leishmaniosis
CI	Confidence interval
ECVD	European College of Veterinary Dermatology
LN	Lymph node
OIF	Oil immersion field
qPCR	Quantitative PCR

## Appendix A

**Table A1:** Sex and neuter status of 20 dogs without CanL (group A), 16 dogs with CanL before treatment (group B), and 17 dogs that were sampled during or after treatment of CanL (group C).

	Group A (*n* = 20)	Group B (*n* = 16)	Group C (*n* = 17)
Intact male	8 (40%)	8 (50%)	6 (35.3%)
Castrated male	1 (5%)	2 (12.5%)	4 (23.5%)
Intact female	4 (20%)	0	4 (23.5%)
Spayed female	6 (30%)	4 (25%)	3 (17.6%)
Missing data	1 (5%)	2 (12.5%)	0

**Table A2:** Age and body weight of 20 dogs without CanL (group A), 16 dogs with CanL before treatment (group B), and 17 dogs that were sampled during or after treatment of CanL (group C).

	Group A (*n* = 20)	Group B (*n* = 16)	Group C (*n* = 17)
Age (years)	7.1 ± 4	7.8 ± 3.4	7.4 ± 2.9
Body weight (kg)	21.8 ± 10.6	13.3 ± 6.2	25.7 ± 12

**Table A3:** Peripheral lymph nodes (LNs) that were sampled from dogs without CanL (group A), dogs with CanL before treatment (group B), and dogs during or after treatment of CanL (group C).

	Group A (*n* = 40 LNs)	Group B (*n* = 32 LNs)	Group C (*n* = 40 LNs)
Popliteal	24 (60.0%)	21 (65.6%)	28 (70%)
Prescapular	10 (25%)	10 (31.3%)	10 (25%)
Submandibular	5 (12.5%)	1 (3.1%)	2 (5%)
Inguinal	1 (2.5%)	0	0

**Table A4:** Size of the peripheral lymph nodes (LNs) that were sampled from dogs without CanL (group A), dogs with CanL before treatment (group B), and dogs during or after treatment of CanL (group C).

	Group A (*n* = 40 LNs)	Group B (*n* = 32 LNs)	Group C (*n* = 40 LNs)
Normal	6 (15%)	6 (18.8%)	4 (10.0%)
Mildly enlarged	15 (37.5%)	13 (40.6%)	23 (57.5%)
Moderately enlarged	11 (27.5%)	12 (37.5%)	8 (20.0%)
Severely enlarged	8 (20%)	1 (3.1%)	5 (12.5%)

**Table A5:** Parasitic burden (logarithmic scale) in peripheral lymph nodes (LNs) of dogs without CanL (group A), dogs with CanL before treatment (group B), and dogs during or after treatment of CanL (group C) when examined twice (1st and 2nd examination round) by three investigators (examiners #1, #2, and #3). Only slides where *Leishmania* amastigotes were found were used to calculate the median and the range.

	Group A (*n* = 40 LNs)	Group B (*n* = 32 LNs)	Group C (*n* = 40 LNs)
Examiner #1			
1st round	1 (1–2)	3 (2–6)	3 (1–6)
2nd round	1 (1–2)	3 (1–6)	3 (1–6)
Examiner #2			
1st round	1	2 (1–5)	3 (1–5)
2nd round	2	2 (1–5)	3 (1–5)
Examiner #3			
1st round	1 (1–1)	3 (1–5)	2 (1–5)
2nd round	1	3 (1–5)	3 (1–5)
